# Evaluation of forensic DNA mixture evidence: protocol for evaluation, interpretation, and statistical calculations using the combined probability of inclusion

**DOI:** 10.1186/s12863-016-0429-7

**Published:** 2016-08-31

**Authors:** Frederick R. Bieber, John S. Buckleton, Bruce Budowle, John M. Butler, Michael D. Coble

**Affiliations:** 1 Center for Advanced Molecular Diagnostics, Department of Pathology, Brigham and Women’s Hospital and Harvard Medical School, 75 Francis Street, Boston, MA 02115 USA; 2 ESR (The Institute of Environmental Science and Research), Private Bag 92021, Auckland, 1142 New Zealand; 3 Statistical Engineering Division, National Institute of Standards and Technology, 100 Bureau Drive, Mail Stop 8980, Gaithersburg, MD 20899 USA; 4 Department of Molecular and Medical Genetics, Institute of Applied Genetics, University of North Texas Health Science Center, 3500 Camp Bowie Blvd., Fort Worth, TX 76107 USA; 5 National Institute of Standards and Technology, Special Programs Office, 100 Bureau Drive, Mail Stop 4701, Gaithersburg, MD 20899 USA; 6 National Institute of Standards and Technology, Applied Genetics Group, 100 Bureau Drive, Mail Stop 8314, Gaithersburg, MD 20899 USA

**Keywords:** Forensic DNA mixtures, Combined probability of inclusion, CPI, Allele drop-out, Stochastic threshold

## Abstract

**Background:**

The evaluation and interpretation of forensic DNA mixture evidence faces greater interpretational challenges due to increasingly complex mixture evidence. Such challenges include: casework involving low quantity or degraded evidence leading to allele and locus dropout; allele sharing of contributors leading to allele stacking; and differentiation of PCR stutter artifacts from true alleles. There is variation in statistical approaches used to evaluate the strength of the evidence when inclusion of a specific known individual(s) is determined, and the approaches used must be supportable. There are concerns that methods utilized for interpretation of complex forensic DNA mixtures may not be implemented properly in some casework. Similar questions are being raised in a number of U.S. jurisdictions, leading to some confusion about mixture interpretation for current and previous casework.

**Results:**

Key elements necessary for the interpretation and statistical evaluation of forensic DNA mixtures are described. Given the most common method for statistical evaluation of DNA mixtures in many parts of the world, including the USA, is the Combined Probability of Inclusion/Exclusion (CPI/CPE). Exposition and elucidation of this method and a protocol for use is the focus of this article. Formulae and other supporting materials are provided.

**Conclusions:**

Guidance and details of a DNA mixture interpretation protocol is provided for application of the CPI/CPE method in the analysis of more complex forensic DNA mixtures. This description, in turn, should help reduce the variability of interpretation with application of this methodology and thereby improve the quality of DNA mixture interpretation throughout the forensic community.

**Electronic supplementary material:**

The online version of this article (doi:10.1186/s12863-016-0429-7) contains supplementary material, which is available to authorized users.

## Background

### Forensic DNA Mixtures

A DNA mixture refers to a biological sample that originated from two or more donors and is determined after a DNA profile is generated. Mixture evidence has always been a part of casework; however there are indications that the fraction of samples presenting as a mixture may have increased, presumably due to changes in methodology, sampling strategies, types of cases (e.g., high volume crime). A retrospective study over a 4 year period in Spain [1] found, in the early years of short tandem repeat (STR) typing, that nearly 95 % of casework samples produced single-source profiles. Initially most mixtures were derived from sexual assault evidence, fingernail cuttings taken by police or at autopsy, from products of conception, and other similar materials. Mixtures from such evidence, combined with the sensitivity of detection of kits at that time, commonly involved only two contributors and one of them (e.g., in sexual assault evidence the person from whom the sample was obtained; in products of conception the biological mother) was “known” and the remaining part of the DNA mixture profile could be inferred to have originated from the second person (i.e., possible person of interest or foreign contributor) [2]. Evaluation of such evidence is, accordingly, comparatively straightforward as the amount of DNA is typically ample, contributions from different individuals are readily evaluated, and the allelic contributions to the DNA evidence of the known individual can be easily “subtracted” from the DNA mixture profile.

In current forensic investigations DNA mixtures occur commonly [3]. Moreover, crime laboratories are being asked to evaluate many more poor-quality, low-template, and complex DNA mixtures. In addition, the forensic community is utilizing methods with an increased sensitivity of detection due to improvements in DNA extraction methods, enhanced multiplex kits, and use of increased number of PCR cycles (or other manipulations) which in turn enable analysis of more challenging and complex mixtures.

At this time, the most commonly used method for forensic evaluation of DNA evidence is the assessment of short tandem repeat (STR) polymorphisms present at multiple distinct genetic loci [4–6]. The amplified products are separated by size using capillary electrophoresis (CE). DNA sequencing also can be used for assessment of STR alleles as well as mtDNA types [7, 8]. After STR analysis, the presence of three or more allelic peaks at two or more genetic loci or peak height differences that are greater than a defined heterozygote peak height ratio are indications that multiple donors contributed to the specific tested DNA sample. A “complex DNA mixture” may contain more than two donors, one or more of the donors may have contributed a low amount of DNA template, or the sample may be somewhat degraded. Low amounts of input DNA will present random (stochastic) effects during DNA amplification on results of STR testing which in turn can lead to failure to detect some or all of the alleles of a true donor (i.e., allele drop-out) [9, 10].

The combined probability of inclusion (CPI) [3, 11, 12] is the most commonly used method in the Americas, Asia, Africa, and the Middle East to assign the weight of evidence where a probative profile is obtained from an evidentiary sample. The CPI refers to the proportion of a given population that would be expected to be included as a potential contributor to an observed DNA mixture. The complement of the CPI is the combined probability of exclusion (CPE). Profile interpretation and CPI calculation involves three steps: assessment of the profile, comparison with reference profiles and inclusion/exclusion determination, and calculation of the statistic.

Prior to comparison with known profiles, peak heights are used to determine whether contributors (i.e., major and minor) can be distinguished. When a known individual’s DNA can reasonably be expected to be present, the known contribution can be “subtracted” [13]. When a known cannot be excluded, the calculation is performed for the evidentiary profile irrespective of any known contributor types, etc.).

The advantages of the CPI approach are thought to be its simplicity and the fact that the number of contributors need not be assumed in the calculation. However, even with simplicity, recently, in the U.S., interpretation protocols used for DNA mixtures using the CPI method have been criticized when applied to forensic mixtures for which it is not suited, highlighting issues of effective communication and technology transfer to the end users of the forensic science community [14]. One should be wary of deceptively simple solutions to complex problems as it is possible that the perceived simplicity of the CPI statistic has led in some instances to incorrect applications of the approach. While the number of alleles is used to generate a CPI statistical estimate, it is incumbent upon the user to evaluate a mixture based on the possible genotypes of the contributors and to consider the potential of missing data (i.e., allele drop-out) based on peak height observations at other loci in the profile and the possibility of allele stacking.

If the DNA crime stain profile is low level, then possibility of allele drop-out should be considered. If allele drop out is a reasonable explanation for the observed DNA results, then the CPI statistic cannot be used at those loci in which the phenomenon may have occurred. The formulation of the CPI statistic requires that the two alleles at each locus of the donor being considered must be above the analytical threshold. Hence, if a profile, or a component of it, is low level, additional considerations are needed to ensure that allele drop-out has not occurred at this locus.

While interpretation of a mixture prior to a statistical calculation requires the direct use of peak heights, the assumed number of contributors, the genotype of known contributors or the genotype of persons of interest (POIs), the CPI calculation, in a strict sense, does not require such consideration [13, 15, 16].

The authors recommend moves in favour of using the likelihood ratio (LR) approaches and laboratories have been embracing LR application [17–19]. Use of the LR also must consider the possibility of allele drop-out; but the LR approach has more flexibility than that of the CPI to coherently incorporate the potential for allele drop-out in complex mixtures (i.e., the so-called probabilistic genotyping methods).

If a lab chooses not to convert to using LRs, or if it does intend to convert but is using CPI in the interim, it remains necessary to ensure that when the CPI is used it is applied correctly.

Herein a more explicit description of a DNA mixture protocol is offered with recommendations for applying the CPI. While the approach described herein overall is not a completely new approach to the use of the CPI, it has become essential to formalize the protocol so that proper statistical analyses can be performed when needed in courtroom proceedings. This protocol is provided as one that should be used for applying the CPI when needed.

Calculation of the CPI involves a statistical model that returns an estimate of the sum of the frequencies of all possible genotype combinations included in the observed DNA mixture. While the computation of the statistical estimate, itself, does not require assumptions about the number of contributors, an assumption of the number of contributors is necessary to help inform decisions about whether allele drop-out is likely at particular loci in the evidentiary sample. For example, if only four allelic peaks appear at a locus in a profile assumed to be from two donors, then it is reasonable to assume that allele drop-out has not occurred at that locus.

That there is no published unifying protocol for use of the CPI for evaluation of forensic DNA mixtures has led to some confusion among forensic practitioners on its proper use. Accordingly a detailed protocol is provided herein to guide the community to reduce variation in interpretation and to promote a more defensible application of the CPI. Three publications describe the use of the CPI [13, 20, 21]. All three of these documents correctly recommend that practitioners should not use (i.e., should disqualify) any locus from the CPI calculation that shows, upon evaluation of the DNA results, that allele drop-out is possible. Moreover, all three support the concept that loci that are omitted for calculation of the CPI statistic may still be used for exclusionary purposes.

Given emerging criticism of methods used in forensic DNA mixture analysis, interpretation and statistical evaluation - particularly in the U.S. - it is timely to revisit and reinforce the foundational principles of interpretation of mixtures and subsequent computation as it relates to the CPI (or CPE). The authors recognize and advocate the community as a whole move towards the use of probabilistic genotyping methods [9, 17, 22, 23] with proper validation. However, in the interim, it has become evident that a specific CPI protocol is needed to guide practitioners who currently use it and for re-analysis of past cases in which use of the CPI method may not have considered the guidelines detailed herein. All methods, including probabilistic genotyping and the CPI-based approach, require the ability to deconvolve mixtures.

It is not possible to prescribe rules for every conceivable situation; therefore, it is essential that application of the CPI be performed by well-trained professionals using their judgement and knowledge under the spirit of the guidelines provided herein, their professional education, and relevant experience. Lastly, the protocol described herein is a guideline and does not preclude alternate acceptable methods to interpret DNA mixture evidence as long as the rules applied are always held subservient to the foundational principles involved in proper mixture interpretation.

## Methods

### Interpretation and application of CPI

Interpretation of a DNA mixture should not be done by simply counting observed alleles. Efforts to deconvolve a mixture into single contributors are advocated where possible [2, 13, 24–26]. If a probative single source profile can be determined at some or all loci then a single-source statistic may be used to calculate a probability estimate (or LR) for that observed profile. This single-source profile may be a deduced major or minor contributor or a deduced foreign contributor by subtracting an assumed known contributor’s alleles.

One caution is that single source statistics at some loci and CPI statistics at other loci should never be combined into one statistical calculation [13]. Either use only those loci that enable a single-source deconvolution or the loci that qualify for a CPI calculation. If the two options are investigated, then the statistic with greatest probative value (i.e., the lower probability of the RMP or CPI) should be reported in order to make optimal use of the data available.

### Rules for qualifying STR loci for use in CPI/CPE calculations on forensic DNA mixtures

The procedure for DNA mixture interpretation using the CPI approach assumes that a laboratory has an established valid analytical (or detection) threshold (AT), stochastic threshold (ST), stutter filter values (SF), and minimum peak height ratio(s). As PCR is “semi-quantitative” STR allelic peak heights are approximately proportional to the amount of DNA from each donor [2, 24]. One might be able to assume that the peak heights may be equivalent at every locus with very pristine (un-degraded) biological samples, but interpretation should be made on the resultant electropherogram [27, 28]. Typically, across an entire DNA profile, there is a downward trend in peak heights such that longer length PCR amplicons, and therefore the alleles contained within, may exhibit shorter peak heights. This phenomenon is referred to as a “degradation slope” (or “ski slope”).

### Impact of the number of contributors on DNA mixture interpretation

DNA mixtures involve two or more donors. It is incumbent upon the DNA analyst to carefully assess and state the assumed number of contributors to a profile, even when using the CPI. The SWGDAM STR Interpretation Guidelines [21] 3.4.1. state “For DNA mixtures, the laboratory should establish guidelines for determination of the minimum number of contributors to a sample.” While we agree generally, the SWGDAM guidelines are not helpful for the evaluation whether allele drop-out may have occurred. An actual number of contributors, not a minimum number, is needed, as a different number of contributors for the same DNA mixture will result in more or less allele drop-out to explain the observed profile. Consider, for example, a mixture profile with exactly 4 alleles at every locus, under the assumption of a two-person mixture there is no evidence of allele drop-out. However, if the assumption is that there are five contributors for the same mixture profile, then probability of allele drop-out is extremely high.

Each donor may contribute 0, 1, or 2 alleles at each genetic marker (locus) tested (with rare occurrences 3 alleles per locus). Any of the observed peaks (true allelic or backward/forward stutter) may overlap with a peak(s) from the same or another donor of the mixture. When allele or artefact sharing occurs there is an additive effect of the two or more peaks, termed “allele stacking” or “allele masking”. As the number of potential contributors increases, so does the uncertainty in accurately determining the true number of contributors [29]. For example, based on the total number of alleles observed across an entire STR profile, it can be extremely difficult, if not impossible, to distinguish a five-person from a six-person DNA mixture and in a number of cases even a three-person from a four-person mixture [29].

These guidelines do not describe in detail how to determine the number of contributors, as a minimum requirement, the number of alleles at each locus and their peak heights should be considered when assigning the number of contributors. Because of the quantity and quality of the DNA being analysed, some loci may meet the determined number of contributors and some may not. For those loci that do not fit the best estimate of the number of contributors, there should be evidence of low signal and/or degradation, which would render the specific locus (or loci) inconclusive for the CPI calculation. Testing additional STR loci may reduce the uncertainty in estimating the potential number of contributors [29]. In addition, challenges arise when close biological relatives have contributed to a mixture or if the DNA is somewhat degraded. Donors to a mixed DNA profile may be referred to as major, minor, and “trace” indicating the relative proportions of their peak heights. For practical purposes minor and “trace” can be considered together as lesser contributors compared with a major contributor(s) of a mixture. In some situations alleles may be missing (i.e., have “dropped out”) in evidentiary samples [30–32].

### Stutter

Stutter, the inherent by-product of slippage during amplification of STRs, adds complexity to mixture interpretation. Typically, interpretation of whether a peak is solely stutter or stutter along with an allele from another contributor arises when a minor or trace contributor peak(s) is observed at a locus (or other loci) that is similar in height relative to the stutter of the major contributor alleles at the locus. These peaks and their heights are used to help determine whether to qualify or disqualify the locus for use in the CPI calculation.

### Stochastic effects

Random variation in peak heights is an inherent property of current DNA typing methodologies. These random variations of peak heights within an individual STR profile or between replicate samples are known as stochastic variation. As the quantity and quality of the input DNA decreases stochastic effects can increase. These effects manifest as variation in peak height between the two peaks at the same locus in a heterozygote or the variation of allele peak heights from the same donor at different loci across the degradation slope line. Such allele peak height variation arises from several factors:

1. Sampling of template from the extract for the aliquot used for the PCR [33],
2. The greater stuttering and lower amplification efficiency of larger alleles (or template accessibility during PCR), and
3. Quality of the template DNA.

It is likely that most of the variation in allele peak heights results from the sampling of template [34, 35] and quality of the sample, but variation during the PCR also contributes, especially with very low template DNA. If the template level is low in the DNA extract then relative variability in the peak heights can be large. This variability is empirically observed and is predicted [36–39]. Because of the strong linear relationship between template (or, more correctly, effective template) and allele peak height, peak height in the actual profile has been a reliable indicator of the presence of stochastic effects and, as such, has been a good indicator for establishing a stochastic threshold (ST) [40, 41].

The ST is the peak height value(s) above which it is reasonable to assume that allele drop-out of a sister allele of a heterozygote has not occurred at a locus [40, 41]. The ST must be determined empirically, based on validation data derived within the laboratory and specific to a given STR kit and analytical instrumentation. Although a binary approach, use of a ST has been deemed important to more formally assess potential allele drop-out. There are several ways in current use to assign a ST (see the Appendix for discussion on setting a ST). A formulaic derivation of the stochastic threshold is displayed in the Additional file 1.

Application of a ST is straightforward for single-source DNA profiles. If a single allele is observed and its peak height is below the ST it is considered possible that a sister allele at that same locus may have dropped out. In contrast to single source samples, in DNA mixtures any given allele peak may actually represent a composite of allele peaks (and depending on position can include stutter peaks). Because of the potential of allele sharing among different contributors to a DNA mixture and the accompanying additive effects in peak heights, a peak height above the ST does not necessarily assure one that a sister allele has not dropped out at that locus. Analysis of the full profile is required to assist in the determination of potential allele drop-out.

Laboratories typically apply a ST for interpretation using a peak height threshold determined based on validation experiments. If the same ST peak height is used across all loci in an entire DNA profile, for many cases involving low level or degraded samples, the loci at the low molecular weight end of the profile (i.e., the smaller amplicons) can exceed the ST whereas at the higher molecular weight end (i.e., the larger amplicons) they may straddle or fall below this threshold.

### Role of STR peak heights and PCR amplification stutter artefacts

STR allelic peak heights are approximately proportional to the effective (i.e., amplifiable) amount of DNA from the donor [2, 24]. Typically, across an entire DNA profile, there is a downward trend in peak heights such that longer sized PCR amplicons, and therefore the alleles contained within them, may exhibit shorter peak heights. Such general peak height behavior and locus-specific performance should be considered in DNA mixture interpretation. The possibility of allele dropout at any particular STR locus is assessed, in part, by use of a ST. The phenomenon of allele drop-out was first documented in the early days of PCR-based typing [10, 42]. Indeed, the Scientific Working Group on DNA Analysis Methods (SWGDAM) recognized the use of a ST and stated in [21] Section 3.2.1: “The RFU value above which it is reasonable to assume that, at a given locus, allelic dropout of a sister allele has not occurred constitutes a stochastic threshold.”

Each STR allelic peak may be associated with one backward stutter peak and occasionally a lower signal forward stutter peak [17, 41–44]. At some loci double backward stutter and “N-2” stutter are observed. Therefore, analysts should be familiar with the nuances of each STR marker. In some situations it may be possible for the stutter peaks from one donor to exhibit a similar height to the allelic peaks from another donor. In such instances the potential allele peaks may not be distinguishable from stutter.

Consider a case where it is ambiguous whether a peak is stutter or an allele. In such an instance a contributor with an allele in this ambiguous position would not be excluded. The appropriate inclusion statistic for this locus then includes the allele probabilities for the ambiguous peak positions in the summation for the CPI calculation [13]. Subtraction of the stutter component may assist in determining the signal from the allelic component of that peak. It might be possible to determine that such peaks must be stutter by assuming a certain number of contributors, or a number of minor contributors. For example, if it is reasonable to assume that there is one minor contributor, and two minor allelic peaks already have been identified, then other small peaks in stutter positions can be assumed to represent true stutter.

## Results and discussion

### Proposed guidelines for an approach to DNA mixture interpretation

The generalized approach is described as follows:

1. Apply a stutter filter as normal and remove any artefacts such as pull-up and spikes.
2. If a single source profile may be deduced from the mixture, then do so. This single-source profile may be a deduced major or minor contributor or a deduced foreign contributor by subtracting an assumed known contributor’s alleles.
  Approaches for calculating single-source statistical estimates of a profile probability can be found in the National Research Council Report [46]. The random match probability (RMP) describes the estimate of the probability that a randomly selected unrelated person would match the deduced single-source (major or minor) profile from the mixture. If a deduced profile is incomplete at any locus (e.g., one obligate allele, but not the other) is deduced, then this uncertainty should be recorded by some nomenclature such as allele “F” or “any” or some other designator. Often the 2p rule is applied for modified RMP calculations at those specific loci [45, 46]. It is reasonable when interpreting a mixture to “subtract” the profile of any donor who could reasonably be expected (or is assumed) to be present in the sample.
3. If no single-source profile could be deduced or there is some interest in interpreting irresolvable components of the mixture, the CPI approach can be invoked.

To formalize the interpretation the overriding principle (P) for use of loci in CPI calculations is:

P_1_: Any locus that has a reasonable probability of allele drop-out should be disqualified from use in calculation of the CPI statistic.

All guidelines that follow are subservient to P_1_. Failing to consider the potential of allele drop- out when there are no detectable peaks between the AT and the ST has allowed the often misguided concept to develop that if all observed peaks are above the ST, then the locus unequivocally can be used.

We cannot prescribe what is a “reasonable probability” as the probability relies on the validation performed by the laboratory and on what ST value has been applied (could be overly conservative). However, if a numerical estimation is sought then one could consider allele drop-out no higher than 0.01 being a reasonable value for addressing uncertainty.

With one exception the approach to DNA mixture interpretation should never trump P_1_. The exception to P_1_ (termed modified or restricted CPI) is an interpretation that can apply to a portion of a profile as opposed to the entire profile. This scenario sometimes occurs where the mixture profile is comprised of multiple major contributors and minor (or trace) contributors where the majors can be resolved readily from the lesser contributing alleles (for example, two major contributors and one minor contributor – (see the section on a major cluster, R_4_) [13, 24, 30].

#### Rule 1 (R_1_) locus qualifying rule

**Fig. 1:**
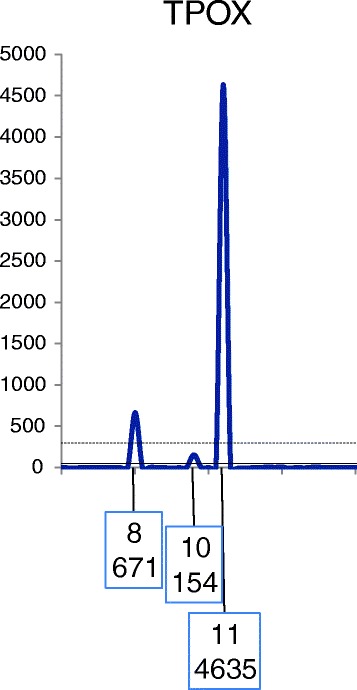
A depiction of the TPOX locus in an assumed two person mixture. Threshold parameters in this example are: ST = 300 and AT = 50 RFU. If the overall profile supports the best assumption of a two-person mixture, then plausible genotype deconvolution should proceed considering a two-person contribution. The ratio of allele 11:8 is ~7:1. If the contributors donated different amounts to the signal, then plausible genotype deconvolutions to explain the mixture are 8, 8 and 11,11 and 8,11 and 11,11. There is little, if any, possibility of the mixture being derived from an “11,11” and an “8,Q” (where Q stands for an unidentified dropped out allele). Hence, there is no reasonable expectation of allele drop-out, and the locus can be used in the CPI calculation

**Fig. 2:**
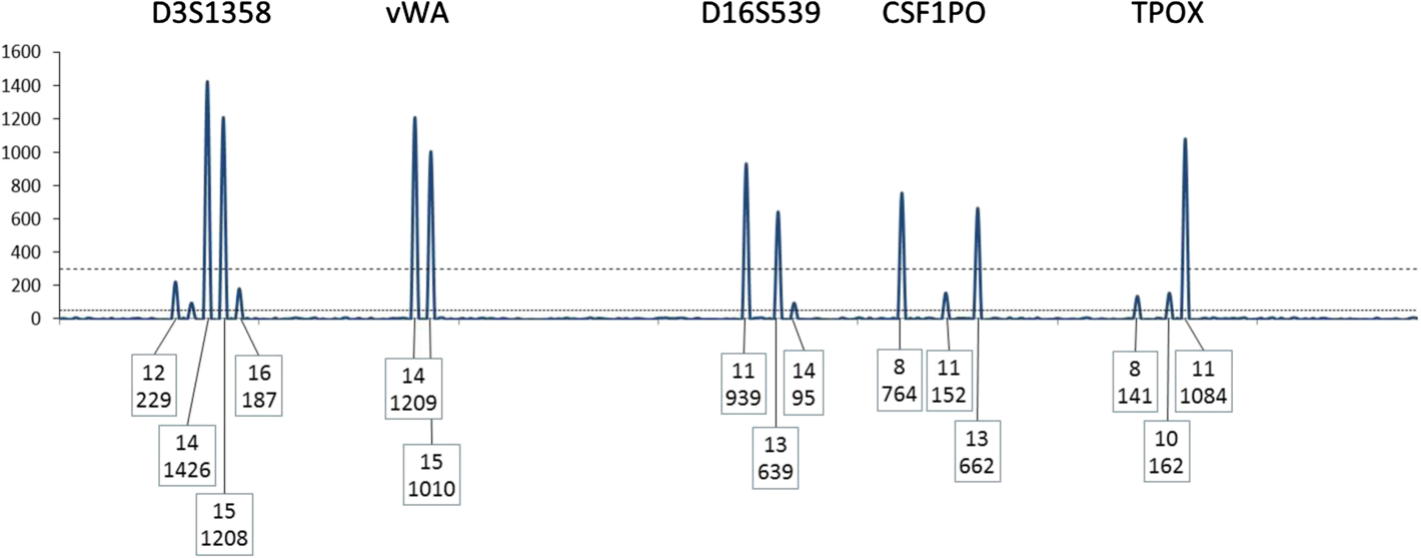
A depiction of the blue dye channel of a Globalfiler STR profile in an assumed two person mixture. Threshold parameters in this example are: ST = 300 and AT = 50 RFU. At four out of five loci there are visible peaks below the ST that can be assigned as alleles and therefore these four loci are disqualified (Rule 1). At the vWA locus no peaks are observed below the ST. However, allele drop-out is possible, suggesting that the vWA locus also should be disqualified from use in the CPI calculation (note the use of Rule 3 below may allow re-qualification of the D3S1358 locus). N.B., as emphasized in the protocol described herein, a major contributor could be determined readily across the entire profile and if attempted all loci would be interpretable for that purpose

A locus is included for use in a CPI calculation if allele drop-out is considered to be highly unlikely. Only qualified loci are used in the calculation of the CPI statistic (Figs. 1 and 2).

Guidance (G) for R_1_.

G_1.1_: Any locus with an allelic peak height below the ST and above the AT is disqualified for a CPI calculation.

For example, as shown in Fig. 2, this Rule would disqualify loci D3S1358, D16S539, CSF1PO, and TPOX (n.b., under the reinstatement rule described below in section R_3_, it may be possible to re-qualify locus D3S1358).

A locus disqualified for a CPI statistic may still be suitable for an RMP calculation.

G_1.2_: Any locus with an observable peak(s) residing below the AT that is likely to be a true allele(s) is disqualified. A peak below the AT may be deemed to be an allele if there is evidence of low level peaks at other loci, the peak(s) is distinct from the local noise, is not in the “N + 4” (i.e., forward stutter) or “N-4” (i.e., backward stutter) or “N-8” (i.e., −2 repeats) stutter position and has Gaussian morphology. While peaks below the AT are not used for comparison purposes, they might be informative to support the possibility of allele drop-out at the locus (or loci) being evaluated, particularly when there are peaks below the ST (and above the AT) at smaller amplicon loci.

G_1.3_: Evaluation of potential allele drop-out is not constrained to observable peaks at a specific single locus. Instead, a global profile evaluation is required. Any locus that has no allelic peaks below the ST and above the AT but may have an unseen allele(s) (based on the peak heights of alleles at other loci) is disqualified.

Implementation of G_1.3_: If there are minor peaks below or close to the ST or below the AT at other loci, these peaks may be indicators of the potential of allele drop-out. These indicator peaks at other loci should be taken into consideration for potential allele drop-out in the specific locus being evaluated.

R_2_: Stutter. Additive effects for alleles overlapping with stutter products must be considered in assessing the potential for allele drop-out at a locus and indistinguishable stutter/allele peaks may need to be included in CPI calculations.

R_2.1_ Check if a peak in a stutter position is considered to have an allele contribution.

G_2.1.1_ To determine whether there is an allele contributing to a peak in the stutter position subtract the stutter threshold or stutter filter value (SF) for the locus from the peak height value for the peak in the stutter position (SPH). The remaining value is the minimum allele contribution (MAC).

SPH – SF = MAC

If MAC > ST, then the locus can be used for use in the CPI calculation.

If MAC ≤ ST, then the locus is disqualified for use in the CPI calculation.

The SF value may not represent the true stutter contribution, as this value often is calculated as the mean stutter + 3SDs. There is a reasonable expectation that the true stutter contribution can be less than the SF value. However, since there is no way to determine the precise stutter contribution, using the maximum value of stutter is advocated.

**Fig. 3:**
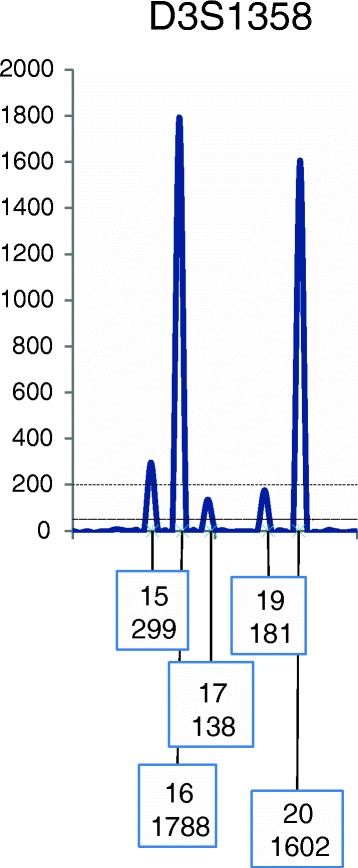
A depiction of the D3S1358 locus showing a two-person profile. Threshold parameters in this example are: ST = 200 and AT = 50 RFU. Using Identifiler Plus data [47], the stutter filter (SF) for the D3S1358 locus is recommended to be set at 12.27 %. The peak height for allele 16 is 1788 RFUs; thus the stutter threshold for a peak at position 15 is 219 RFUs. The observed peak height at position 15 is 299 RFUs. Therefore, the MAC is 80 RFUs (i.e., 299-219 = MAC). Since 80< ST, the potential for allele drop-out is invoked, and the locus would be disqualified. However, if the overall profile interpretation supports a single minor contributor, then the contributing allele at position 15 can be paired with the minor obligate allele 17 (138 RFUs), and the locus now can be re-qualified (see exception rule R_3_), even though both minor allele peak heights are below the ST. While using SWGDAM and ISFG guidelines [18, 19, 21] this example a major profile should be deconvolved, for demonstration purposes a CPI calculation is shown using alleles 15,16,17,20 (the peak at 19 is assumed to represent stutter). R_2.2_ If there is a minor allele of approximately the height of a possible allelic component of a stutter peak and there is at least one minor allele unconfirmed then the stutter peak(s) should be included in the summation for the CPI calculation (Figs. 3 and 4)

G_2.1.2_ The locus may be re-qualified (see exception rule R_3_ below) even when the MAC ≤ ST, if there is evidence of no allele drop-out at the locus. Evidence of no allele drop-out could come from a deconvolution where all minor or trace alleles have been observed or inferred based on subtraction of an assumed known contributor’s alleles. Determining the number of minor contributors (and hence the number of possible minor alleles) can be challenging with complex DNA mixtures. A peak in the stutter position that does not exceed the SF may still have been comprised of both stutter and an allele from another contributor. This peak(s) should be considered potentially allelic based on the data in the profile (Fig. 3).

**Fig. 4:**
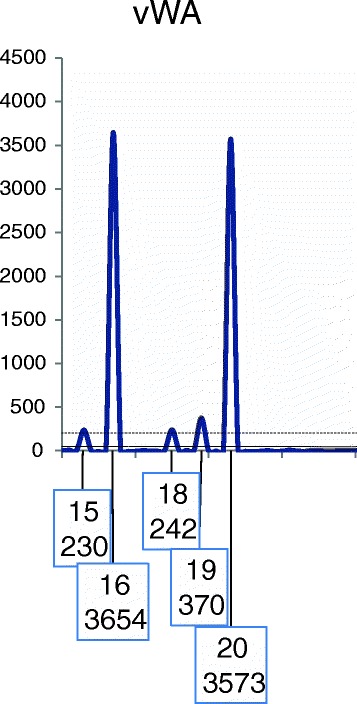
A depiction of the vWA locus illustrating the application of R_2_. Threshold parameters in this example are: ST = 200 and AT = 50 RFU. Hence the obligate minor allele at 18 is above the ST and drop-out of its sister allele is unlikely. This locus is qualified for use in the CPI calculation. Under the assumption of two contributors there is one minor allele unconfirmed. Both the 15 and 19 peaks are below the stutter filter (SF) and hence could be all stutter or a composite of stutter and allele. This example illustrates the scenario where peaks in the stutter position fall below the SF. The partner to the 18 allele must be at one of the positions 15,16,18,19, or 20. Since the minor contributor genotype cannot be resolved with sufficient confidence, for this example the probability of inclusion is calculated as *PI* = (*p*_15_ + *p*_16_ + *p*_18_ + *p*_19_ + *p*_20_)^2^

R_2.3_ If there is no minor allele at this locus but other loci suggest that the height of a possible minor allele at this locus is approximately the height of a peak in a stutter position, the stutter peak(s) should be included in the summation for the CPI calculation.

### R_3_: exception rule. Indicators that alleles below the ST did not drop-out

It is possible to reinstate (requalify) some loci for use in the CPI calculation. This qualification can occur for alleles observed at a locus, dependent on the assumption of the number of contributors to that mixture even where the peak height of an allele(s) falls below the ST (and above the AT). As stated above, while the number of contributors is not taken into account when calculating the CPI, it is imperative that the number of contributors be assumed to determine the potential of allele drop-out. For example, consider a two-person mixture with one major and one minor contributor (Fig. 1), and the assumption of one minor contributor reasonably can be made. If two minor alleles are observed, then the locus may be used in the CPI calculation, regardless of whether any of the minor alleles are below the ST. In this scenario (and other similar ones) there is no indication of allele drop-out at the locus. Referring back to Fig. 2, this qualification would reinstate the D3S1358 locus and allow its use in a CPI calculation.

This approach can be extended to three-person mixtures if interpretation of the overall profile indicates that allele drop-out has not occurred under an assumed number of contributors.

G_3_: If a mixture interpretation suggests no drop-out, then the locus can be used in the CPI calculation.

G_3.1_: If all possible alleles are observed (e.g., a two-person mixture and 4 alleles), then the locus can be used in the CPI calculation.

#### R_4_: major cluster rule

**Fig. 5:**
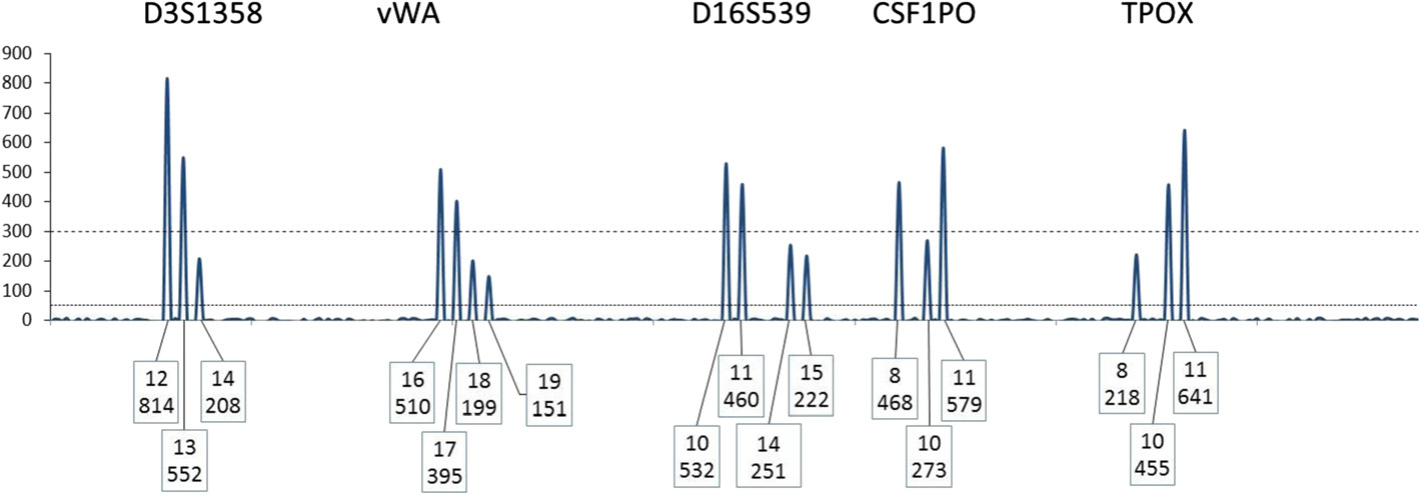
A depiction of a hypothetical depiction of the blue dye channel of a Globalfiler electropherogram in an assumed two person mixture. Threshold parameters in this example are: ST = 300 and AT = 50 RFU. A POI who is 13,13 at D3S1358 would support an exclusion with two assumed contributors. If this POI were included then the other contributor would have to be 12, 14 at the locus with an improbable PHR

If a set of peaks representing more than one donor is distinct from one or more minor or trace peaks then the CPI approach may be applied to the “major cluster” (see G_4.1_, Fig. 5, Table 1). We outline an algorithm to confirm a major cluster (see Appendix).

**Table 1:** The peak height analysis for the STR profile shown in Fig. 6

Locus	*SMP*	*LTP*	$\frac{LTP\left(2NT-T\right)}{PHRL}$	Pass/fail Major cluster	$SMP-\frac{LTP\left(2NT-T\right)}{PHRL}$	
D3S1358	1698	290	580	Pass	1118	Qualified
vWA	1648	289	578	Pass	1070	Qualified
D16S539	1386	336^a^	672	Pass	714	Qualified
CSF1PO	1380	206	412	Pass	968	Qualified
TPOX	1727	289	578	Pass	1149	Qualified

*SMP* smallest main peak, *LTP* largest trace peak, *NT* number of trace contributors, *T* number of trace alleles, *PHRL* peak height ratio limit value. ^a^The “9” peak at D16S539 may be larger because of a stutter component. Hence LTP is 336 RFU or less

G_4.1_: To qualify a locus for use with a major cluster, first there must be a clear visual distinction between a set of large peaks and a set of trace peaks. The principle is that all major peaks must be identifiable and for these major peaks allele drop-out must be deemed unlikely.

There are two aspects to this principle;

- G_4.1.1_ Any allele peak assigned to the major cluster must be sufficiently high that it could not have a partner allele in the minor set, and
- G_4.1.2_ Allele peaks assigned to the major cluster must be sufficiently high that allele drop-out is unlikely even when consideration is given that the peak might be a composite of major and minor.

**Fig. 6:**
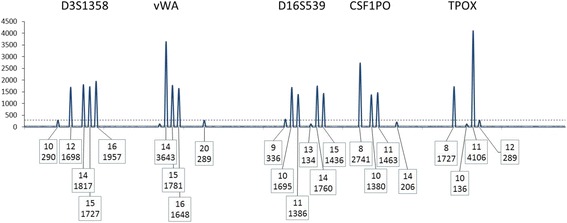
A depiction of the blue dye channel of a Globalfiler electropherogram. Threshold parameters in this example are: ST = 200 and AT = 50 RFU. This example is an acceptable “major cluster”. There is one trace contributor (NT = 1). For this example a peak height ratio limit (PHRL) of 0.50 is used (See Table 1 for peak height analysis using the major cluster rules). The PHRL should be determined by each laboratory based on validation studies

**Fig. 7:**
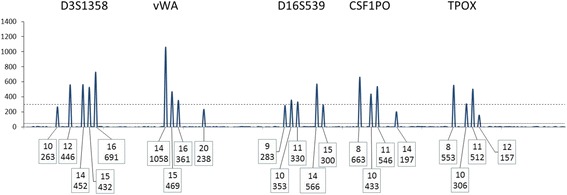
A depiction of the blue dye channel of a Globalfiler STR profile. Threshold parameters in this example are: ST = 300 and AT = 50 RFU. This example is an unacceptable major cluster. There is one minor contributor (NT = 1). The two major profiles are not much greater in height than the minor profile

**Fig. 8:**
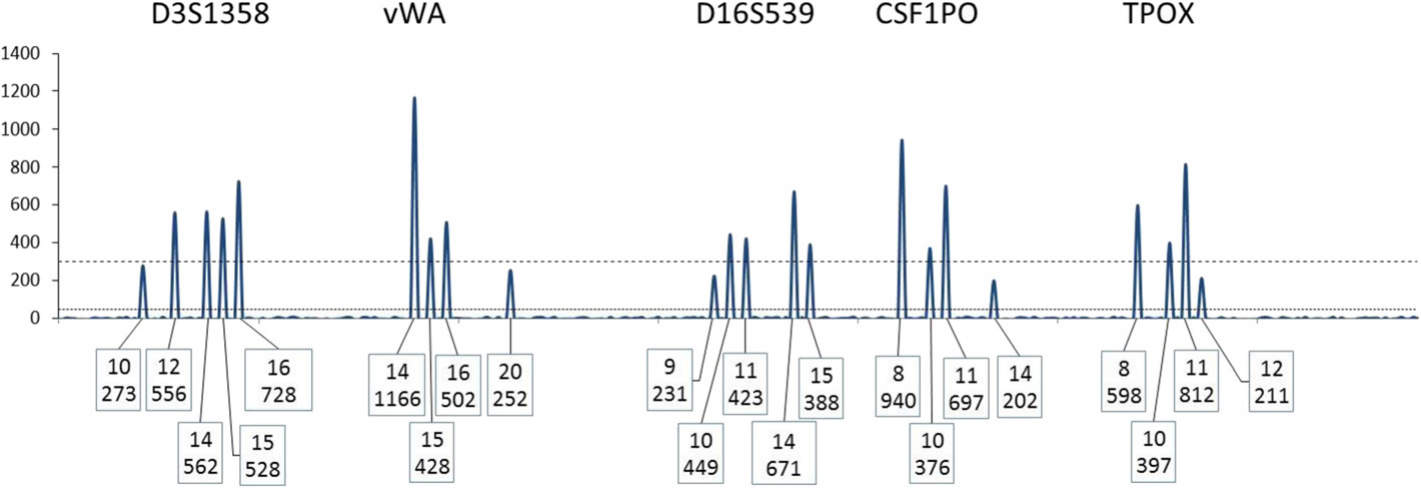
A depiction of the blue dye channel of a Globalfiler electropherogram. Threshold parameters in this example are: ST = 300 and AT = 50 RFU. This example is intended to illustrate an unacceptable major cluster. There is one minor contributor (NT = 1). The two major profiles look to be about twice the height of the minor. A PHRL of 0.50 is used for this example. See Table 2 for the peak height analysis using the major cluster rule

G_4.2_: This assessment requires some level of deconvolution and is more straightforward if there are only two major profiles and one trace contributor. Consider a locus with four large peaks and two small ones (Fig. 6). Such a profile (at this single locus) is consistent with being from two major profiles and one trace profile. In such a case determine that a trace peak and a major peak cannot be misassigned. If there are only three, two, or one major peaks present, check that any peaks assigned as trace could not be a major peak. This approach is best accomplished by visualizing the major and trace peaks across the entire profile and fitting realistic degradation curves. If there is no distinction between a set of large peaks and the small ones at a locus (or loci), then assigning a “major cluster” should not be attempted (Figs. 7 and 8).

#### R_5_. calculation of CPI/CPE

The formula for calculating the CPI has been described elsewhere [10] (Appendix). For each of the qualifying loci sum the allele frequencies for the allelic or potentially allelic peaks (those peaks added by the stutter rule) and square that value. Multiply the value of each locus that qualified under the assumption of independence to produce the CPI (n.b., the CPE is 1-CPI).

G_5.1_ With the exception of using data from a reference profile in which an assumption of one of the contributors is known, such as from an “intimate” sample (described in G5.3), comparison of a DNA mixture profile with that of a known suspect/victim or other known POIs, when possible, should not be carried out until the mixture evidence has been fully evaluated as described above. Comparison of the evidence and known profiles for inclusion/exclusion purposes is independent of the CPI statistical calculation. Regardless, All the alleles of the POIs should have a corresponding allelic or potentially allelic peak in the qualifying loci. If the evidence supports an exclusion, the calculation of the CPI is unnecessary for that comparison. If there is a failure to exclude based on genotype possibilities derived from peak heights at qualified or disqualified loci, then a computation is provided. Computation of the CPI does not require examination of the STR profile (genotypes) of the known individuals (suspect, victim, POIs). At the point of computation of the CPI, the DNA mixture profile is composed of qualified and disqualified loci.

G_5.2_ There can be only one value for the CPI/CPE computed for each DNA mixture profile. The interpretation of potential allele drop-out should be made prior, when possible, to evaluating known reference samples. Adjustments to fit the interpretation to reject or reinstate a locus based on additional information from a person of interest profile (i.e., confirmation bias and fitting the profile interpretation to explain missing data based on a known sample) are inappropriate [48–50].

G_5.3_ One exception to using data from a reference profile is where an assumption of one of the contributors is known, such as from an “intimate” sample. The assumption of the individual(s) being a known contributor(s) must be documented. In situations where a contributor(s) is assumed, then subtraction of the alleles of the known contributor(s) is justified (which also may be applied to major cluster analyses).

G_5.4_ Use of the 2p rule for the CPI is not valid.

The 2p statistic was designed for single-source samples where one allele was present at a locus and there was strong support for allele drop-out.

### Exculpatory evidence

Once the mixture has been evaluated, both the qualified and disqualified loci should be inspected for potential exclusionary evidence. For the qualified loci exclusionary evidence may be based on the absence of alleles or the absence of deconvolved genotypes in the mixture compared with those of the known reference profile. If the deconvolved genotypes of the mixture are different from the genotype of the known comparison profiles, then an exclusion interpretation is supported. If the locus (or loci) was deemed disqualified for the CPI calculation, allele drop-out should be considered when including or excluding a potential donor.

R_6_. For the qualified loci exclusionary evidence exists when the POI has any alleles not present in the crime stain profile.

Consider the D3S1358 locus shown in Fig. 2. The rest of the profile supports a two-person mixture. Initially this locus would be disqualified based on having peaks below the ST but then is reinstated because both minor peaks are present. At this locus a POI is excluded if the POI has any allele outside the set [12, 13, 15, 16].

R_7_. For loci that can be deconvolved exculpatory evidence exists when the POI has a genotype not amongst the set of supported genotypes.

Consider again the D3S1358 locus (Fig. 2). At this locus a POI is excluded if the POI has a genotype other than the genotypes {12,16 or 14,15}.

R_8_. For disqualified loci exculpatory evidence can occur but relies on the profile, allowing for missing data, to determine if the POI is unlikely to be a donor.

G_8_. The POI is unlikely to be a donor if the allele(s) consistent with the POI and the total number of observed alleles at a given locus invalidate or do not support the assumed number of contributors to the DNA mixture. The inclusion of the POI would cause a mismatch with the assumed number of contributors (Fig. 5). Before finalizing an exclusion ensure that the assumed number of contributors holds throughout the profile. If that assumption is not valid, the result may be considered inconclusive.

## Conclusions

### The path forward

The protocol described herein is intended to help reduce confusion and misunderstanding in the forensic community about how to best apply the CPI in evaluation of forensic DNA mixtures, not only for current casework but for retrospective review of past cases. While the protocol detailed herein is not novel in the sense that most aspects of the CPI have been discussed in the literature, the lack of a unifying detailed CPI protocol has led to confusion and in some cases misapplication of this method. For this reason it is important that a detailed DNA mixture interpretation protocol be offered to reduce inter- and intra-laboratory variation in application of the CPI. Cases for which a CPI was calculated without considering the possible presence of allele drop-out or other stochastic effects might benefit from a thorough scientific review. Other cases for review could include those in which multiple CPIs were computed on the same mixture profile, or when confirmation bias was possible (e.g., when “suspect-driven” mixture analysis was performed).

In Texas, the Forensic Science Commission has been working with laboratories to assess the DNA mixture protocols and review the statistical analyses in selected cases using the CPI/CPE. For laboratories or jurisdictions that modify their DNA mixture interpretation protocols, either in light of this document or for other reasons, there may be reason to review a sample of selected pending or previously reported DNA mixture casework. Forensic laboratories can work closely with all stakeholders in their respective jurisdictions to address these issues in a collaborative and constructive manner.

## Appendix

### Determination and use of stochastic thresholds

Several approaches have been used to determine stochastic thresholds. These include:

1. Methods based on largest surviving allele,
2. Methods based on peak height ratio studies, and
3. Methods based on assigning a probability of drop-out, Pr (D).

We have not specifically tested these different methods against each other and hence do not recommend one method over the other. There is a compromise required when setting the ST: The higher that it is set the lower the risk that dropout is actually possible, but the more information that is wasted.

The ST must be empirically determined based on data derived within the laboratory and specific to a given amplification kit and the detection instrumentation used? The laboratory should evaluate the applicability of the ST among multiple instruments (i.e., is one CE more sensitive than others?). If measures are used to enhance detection sensitivity (e.g., increased amplification cycle number, increased injection time), the laboratory should perform additional studies to establish a separate stochastic threshold(s).

1. Methods based on the largest surviving allele
  In this method a study is made of DNA samples constructed from known donors so that the genotypes of the input DNA are known with certainty (known ground truth). Often these samples are pristine and single source. Input DNA amounts that span the range over which allele “drop-out” is expected are amplified. Loci where the known ground truth is a heterozygote profile and where one allele has dropped out are noted and the height of the surviving allele is recorded. The ST is placed at some position with regard to the height of the “surviving allele”. This placement often is the maximum peak height observed.
  While useful, the surviving allele method does not directly address the probability of allele drop-out at the ST. This method of determining the ST is limited by sampling, such that the larger the number of samples, the greater is the chance of observing allele drop with a higher surviving partner peak height. Consider that a value for ST is chosen from a dataset of size N such that allele drop-out has never been observed with a surviving allele higher than this threshold. It is tempting to conclude that at this ST the probability of allele drop-out is zero. In reality, if N is deemed large (e.g., 100 different profiles in the stochastic range), then the probability of allele drop-out at this ST will be small but likely not zero. In contrast, if a much larger sample (e.g., *N* = 1000) is used there will be a possibility of some surviving alleles with dropped allelic (“sister allele”) partners above the previous ST.
2. Methods based on peak height ratio studies
  In this method of implementing a ST, the same type of data as described above can be used. It is valuable to analyze down to below the AT that will be used in casework as this analysis helps with the average peak height (APH) for low level data. For example, if it is proposed to use 50 RFUs as an AT in forensic casework, then it may be suitable to analyze samples down to as low as 20 or 25 RFUs (the “research AT”). The peak height ratio (PHR) and the APH for each heterozygote locus then is determined. Missing data (alleles that have dropped out below the research AT) are input at some value (e.g., half the research AT) to determine the APH and PHR. A plot then is made of PHR vs APH. A curve (the peak height ratio limit, *PHRL*) is fitted to these data of the form $PHRL=\frac{k}{\sqrt{APH}}$ that captures all or most of the data. This value should be set to capture 0.995 of the data. This setting can be done simply by plotting the line $PHRL=\frac{k}{\sqrt{APH}}$ on the graph of *PHR* v *APH* and varying the value for *k*. Once *k* is assigned then $\log \frac{ST}{AT}=\frac{k}{\sqrt{\frac{AT+ST}{2}}}$. This equation has no algebraic solution and has to be solved by numerical means. The probability of dropout when the surviving peak is at the ST is approximately the fraction of the data not captured by the fitted curve (using the recommended value of 0.995 this is 0.005). This method can explicitly obtain the probability of allele drop-out.
3. Methods based on assigning a probability of drop-out, Pr (D)
  In this method of ST placement the same type of data as described above can be used. As stated above, it is valuable to analyze down to below the AT that will be used in forensic casework. The method described in [33] is used to calculate a function giving the probability of allele drop-out and produces constants 0 and 1. If *α* is the probability of allele drop-out accepted by the laboratory for the ST (e.g., 1 in 1000) then $\mathrm{S}\mathrm{T}={e}^{\frac{ \ln \left(\frac{\alpha }{1-\alpha}\right)-{\beta}_0}{\beta_1}}$ where *β*_0_ and *β*_1_ are coefficients from the logistical regression. Timken and colleagues discuss use of a closely similar approach [31].
  If the ST is applied as is typically done (i.e., an allele above the ST is assumed to have a partner that has not dropped out), then the probability of allele drop-out is technically larger by an unknown amount. This expectation is because the probability of allele drop-out is assigned from the expected height of peaks at this locus based on the entire profile across all loci, and not simply the height of one allele peak.
  CPI/CPEThe inclusion probability also can be defined as: the probability that a random person would be included as a contributor to the observed DNA mixture. The complement of the *CPI* is the combined probability of exclusion (CPE). It proceeds in two steps, an inclusion/exclusion phase followed by the calculation of a statistic. When a person of interest is not excluded then: If the mixture has alleles *A*_*1*_*… A*_*n*_ then the inclusion probability at locus *l*, (*PI*_*l*_) is $P{I}_l={\left({\displaystyle \sum_ip\left({A}_i\right)}\right)}^2$ if Hardy-Weinberg Equilibrium expectations are assumed. By writing ${\displaystyle \sum_ip\left({A}_i\right)}=p$ the *PI*_*l*_ = *p*^2^ is obtained.
  The *PI* across multiple loci (*CPI*) is calculated as $CPI={\displaystyle \prod_lP{I}_l}$

### A suggested algorithm for confirming a major cluster

The following algorithm is based on a valid peak height ratio limit value, PHRL. Determine the largest trace peak (*LTP*) and number of the minor or trace contributor(s) at that locus (*NT*). This evaluation should be done by considering all minor or trace peaks at this locus along with any indicator peaks (trace alleles) at other loci. Apply a plausible degradation curve to the profile if needed. Check that LTP is not low with regard to the trace peaks at other loci. If it is, one can adjust its height upwards.

One way to qualify a locus for use as a “major cluster” is to consider the smallest major peak (SMP):

The sum of the heights of all unseen trace peaks (2NT-T), where T is the number of trace peaks observed, is not expected to exceed the value computed by $\frac{LTP\left(2NT-T\right)}{PHRL}$.

If $SMP>\frac{LTP\left(2NT-T\right)}{PHRL}$ then this peak must have a component from a major contributor in it.

Check that this component is large enough that allele drop-out is unlikely. This assumption of no allele drop-out is expected to be true if the smallest major component exceeds the *ST*. Test this assumption by application of the inequality $SMP-\frac{LTP\left(2NT-T\right)}{PHRL}>ST$ otherwise the locus is disqualified.

If *T* = 2NT then $$ SMP>\frac{LTP}{PHRL} $$

If the *SMP* is small (e.g., less than *ST*) it is likely that the PHR is too large and the formulas cannot be relied upon (Figs. 6 and 7, Table 2). While these specific rules have not been described in detail (although inferred in [12]) they may appear novel. However, they derive deductively from the PHR. The validity of this rule relies on the validation of the laboratory’s PHR.

**Table 2:** The peak height analysis using the major cluster rule for the STR profile shown in Fig. 8. A visual inspection alone should suggest that a major cluster cannot be assigned for this profile since there is no clear separation between a set of large peaks and smaller ones. However the analysis is performed for demonstration purposes

Locus	Major cluster peaks	SMP	*LTP*	$\frac{LTP\left(2NT-T\right)}{PHRL}$ or $\frac{LTP}{PHRL}$	Pass/fail	$SMP-\frac{LTP\left(2NT-T\right)}{PHRL}$
D3S1358	Top four	528	273	546	Fail	All terms in this column are negative.
vWA	Top three	428	252	504	Fail	
D16S539	Top Four	388	231	462	Fail	
CSF1PO	Top three	376	202	404	Fail	
TPOX	Top three	397	211	422	Fail	

## Additional file

Additional file 1: A Supplemental Materials section is provided which shows a formulaic derivation of the stochastic threshold. (DOC 251 kb)

## Abbreviations

AT: Analytical threshold
CE: Capillary electrophoresis
CPE: Combined probability of exclusion
CPI: Combined probability of inclusion
LR: Likelihood ratio
MAC: Minimum allele contribution
mtDNA: Mitochondrial DNA
PCR: Polymerase chain reaction
POI: Person of interest
RFU: Relative fluorescent unit
RMP: Random match probability
SF: Stutter filter value
SPH: Peak height value in the stutter position
STRmix^TM^: Forensic software
STR: Short tandem repeat
ST: Stochastic threshold
SWGDAM: Scientific working group on DNA analysis methods
