# Supplementary material for: Evaluation of forensic DNA mixture evidence: protocol for evaluation, interpretation, and statistical calculations using the combined probability of inclusion
Source: BMC Genet. 2016 Aug 31;17(1):125. doi: 10.1186/s12863-016-0429-7 (PMC5007818; doi:10.1186/s12863-016-0429-7)
Supplement: Additional file 1: — A Supplemental Materials section is provided which shows a formulaic derivation of the stochastic threshold. (DOC 251 kb) [file 12863_2016_429_MOESM1_ESM.doc]

**Supplementary Note:**

**Derivation of ST**

Derivation of $\log\frac{ST}{AT}=\frac{k}{\sqrt{\frac{AT+ST}{2}}}$

$$\log\left(PHRL\right)=\frac{k}{\sqrt{APH}}$$

The biggest possible PHR with one peak at ST is $\log\frac{ST}{AT}=\log\left(PHRL\right)=\frac{k}{\sqrt{APH}}=\frac{k}{\sqrt{\frac{AT+ST}{2}}}$

🡪 $\log\frac{ST}{AT}=\frac{k}{\sqrt{\frac{AT+ST}{2}}}$

The logistic equation is $\Pr(D)=\alpha=\frac{e^{\beta_{0}+\beta_{1}\log(ST)}}{1+e^{\beta_{0}+\beta_{1}\log(ST)}}$, where Pr(D) = probability of allele drop-out
